# Accurate and Scalable Quantum Hydrodynamic Simulations of Plasmonic Nanostructures Within OFDFT

**DOI:** 10.3390/nano15161288

**Published:** 2025-08-21

**Authors:** Qihong Hu, Runfeng Liu, Xinyu Shan, Xiaoyun Wang, Hong Yang, Heping Zhao, Yonggang Huang

**Affiliations:** 1 College of Physics and Electromechanical Engineering, Jishou University, Jishou 416000, China; 2 School of Information Technology and Management, Hunan University of Finance and Economics, Changsha 410205, China

**Keywords:** quantum hydrodynamic theory, orbital-free density functional theory, energy functional, plasmonic nanostructure

## Abstract

Quantum hydrodynamic theory (QHT) provides a computationally efficient alternative to time-dependent density functional theory for simulating plasmonic nanostructures, but its predictive power depends critically on the choice of ground-state electron density and energy functional. To construct ground-state densities, we adopt orbital-free density functional theory and numerically evaluate the effect of different exchange–correlation functionals and kinetic energy functionals. A suitable energy functional to reproduce both the DFT-calculated work function and charge density is identified. In the excited-state part, we adopt this obital-free ground-state density and investigate how variations in the von Weizsäcker kinetic energy fraction within the Laplacian-level functional affect the resonance energy and oscillator strengths. The appropriate functional form is identified, achieving an accuracy comparable to that reported in previous studies. Applied to sodium nanodimers, our approach captures nonlinear density responses at sub-nanometer gaps. This work extends QHT beyond idealized geometries and offers a robust path toward efficient quantum plasmonic modeling.

## 1. Introduction

Plasmonic nanostructures have attracted significant attention due to their ability to localize and enhance electromagnetic fields at the nanoscale, enabling a broad range of applications in sensing, catalysis, energy harvesting, and quantum optics [1,2,3,4,5,6,7]. When the physical dimensions of these structures are reduced to a few nanometers, or when interparticle or interfacial gaps shrink below the nanometer scale, quantum mechanical effects become increasingly important, and classical electrodynamics fails to capture key physical phenomena [8,9,10,11,12]. Time-dependent density functional theory (TDDFT) provides a formally exact framework for describing electronic excitations, accurately capturing both many-body interactions and quantum effects [13,14]. However, its computational cost scales steeply with system size, limiting its practical use to relatively small systems containing only thousands of electrons [15,16,17]. To address the modeling needs of larger and more realistic systems, several approximate theoretical frameworks have been developed. The random phase approximation captures collective electron oscillations and has been successfully applied in analyzing energy transfer and quantum plasmonic effects [18,19,20]. Feibelman’s d-parameter method introduces quantum surface corrections into Maxwell’s equations via effective boundary terms, allowing efficient integration with classical electrodynamics [21,22]. Quantum hydrodynamic theory (QHT), which has recently seen substantial progress in modeling surface plasmonic effects of nanostructures, offers another promising alternative [23,24,25,26,27,28].

QHT offers a favorable trade-off between accuracy and efficiency, retaining the ability to capture key quantum phenomena—such as spatial nonlocality and charge spill-out—which are particularly important in systems with narrow gaps and small particle sizes [8,9,10,11], at a fraction of the cost of TDDFT. However, for QHT to be predictive and generalizable to realistic nanostructures, several fundamental challenges remain unresolved. First, current ground-state density inputs either rely on computationally expensive density functional theory (DFT) calculations or adopt simplified analytical models that cannot be generalized to arbitrarily shaped systems [23,24]. Although orbital-free (OF) DFT provides a potentially efficient solution, energy functionals suitable for QHT have not been systematically studied [25,26,28]. In particular, existing works employ diverse local density approximation (LDA) exchange–correlation (XC) functionals, and the von Weizsäcker (vW) kinetic energy weight $\lambda_{W}$ without a consistent rationale, leaving the impact of these choices unclear. Second, in the excited-state calculations, the choice of $\lambda_{W}$ also significantly affects the accuracy of predicted plasmonic resonances and thus requires careful calibration [23,28]. Third, for multiparticle systems such as nanodimers, the commonly used linear superposition of monomer densities remains an approximate treatment whose validity at sub-nanometer separations is questionable and requires critical assessment [24].

In this work, we develop OF-PGSLN, a self-consistent and generalizable QHT framework that integrates OFDFT with Laplacian-level kinetic energy functionals to overcome these challenges. Our development proceeds in two parts. In the ground-state part, we generate electron densities directly from OFDFT and investigate how different XC functionals and the vW term weight $\lambda_{W}$ influence the density profile and work function. These comparisons demonstrate that identifying suitable energy functionals within the OFDFT framework is both important and meaningful for QHT applications. In the excited-state part, we benchmark the QHT response using a single sodium nanosphere and examine how variations in $\lambda_{W}$ affect the computed plasmonic resonance. Compared to Ref. [24], which employed analytical densities and fixed $\lambda_{W}=1$, our framework maintains similar accuracy while offering a more flexible and physically grounded approach to generating ground-state densities. Furthermore, we apply OF-PGSLN to sodium nanodimers and show that the commonly adopted linear superposition method fails to reproduce the nonlinear density redistribution and plasmonic shifts at sub-nanometer separations, whereas our method captures these effects reliably.

In summary, this work advances the field of quantum hydrodynamic modeling by presenting a fully self-consistent, numerically stable, and extendable framework. By addressing key limitations of earlier approaches, OF-PGSLN moves QHT one step closer to practical application in quantum plasmonics. The remainder of this paper is organized as follows: Section 2 introduces the theoretical formulation; Section 3 discusses the ground-state density calibration; Section 4 benchmarks the optical response; Section 5 applies the method to nanodimer systems; and Section 6 concludes this paper.

## 2. Model and Method

The conventional linearized QHT response is governed by the following equations in the frequency domain [23,24,25,26,27,28]:

(1) $$\nabla \times \nabla \times E_{s}-\frac{\omega^{2}}{c^{2}}E_{s}=\omega^{2}\mu_{0}P,$$

(2) $$\frac{en_{0}}{m_{e}}\nabla {\left(\frac{\delta G[n]}{\delta n}\right)}_{1}+\left(\omega^{2}+i\gamma \omega \right)P=-\varepsilon_{0}\omega_{p}^{2}\left(E_{i}+E_{s}\right).$$

Here, $E_{s}$ is the scattered field and $E_{i}$ is the incident field. In this work, plane waves incident perpendicular to the *z*-axis are used. *c* is the speed of light in vacuum. $\varepsilon_{0}$ and $\mu_{0}$ are the vacuum permittivity and permeability. $m_{e}$ and *e* are the electron mass and charge (in absolute value). γ represents the phenomenological damping rate. In this paper, we choose 0.22eV, which can provide corresponding oscillator strength in line with TDDFT [24]. The plasma frequency $\omega_{p}\left(r\right)=\sqrt{e^{2}n_{0}/\left(m_{e}\varepsilon_{0}\right)}$ depends on the spatially dependent ground-state (equilibrium) electron density $n_{0}$. The quantity ${\left(\delta G[n]/\delta n\right)}_{1}$ can be obtained by adopting a first-order perturbation approach, where the perturbed density is taken as $n\left(r\right)=n_{0}\left(r\right)+n_{1}\left(r\right)$, with $n_{1}\left(r\right)=\nabla \cdot P/e$ being a small (by assumption) first-order dynamic perturbation. The energy functional G[n] is given by

(3) $$G\left[n\right]=T_{s}\left[n\right]+E_{\mathrm{XC}}\left[n\right].$$

Here, $E_{\mathrm{XC}}\left[n\right]$ is the XC functional, while $T_{s}\left[n\right]$ is the noninteracting kinetic energy functional. Most recently [24], the noninteracting kinetic energy functional depending on the Laplacian of the electronic density has been proposed, which has the form

(4) $$T_{s}\left[n\right]=\int \tau \left(n,w,q\right)\;d^{3}r,$$

with $w={\left|\nabla n\right|}^{2}$ and $q=\nabla^{2}n$. The kinetic energy density τ(n,w,q) is approximated as the sum of the vW $\tau^{\mathrm{vW}}\left(n,w\right)$, the Pauli–Gaussian (PGα) $\tau^{\mathrm{PG}\alpha }\left(n,w\right)$, the Laplacian (Lβ) $\tau^{L\beta }\left(n,q\right)$, and a modified term $\tau^{Nq_{0}}\left(n,q\right)$:

(5) $$\tau \left(n,w,q\right)=\tau^{\mathrm{vW}}\left(n,w\right)+\tau^{\mathrm{PG}\alpha }\left(n,w\right)+\tau^{L\beta }\left(n,q\right)+\tau^{Nq_{0}}\left(n,q\right),$$

where

(6a) $$\tau^{\mathrm{vW}}\left(n,w\right)=An^{-1}w,$$

(6b) $$\tau^{\mathrm{PG}\alpha }\left(n,w\right)=\tau^{\mathrm{TF}}\left(n\right)\;e^{-\alpha Cn^{-8/3}w},$$

(6c) $$\tau^{L\beta }\left(n,q\right)=\beta Dn^{-5/3}q^{2}=\beta \tau^{\mathrm{TF}}\left(n\right)q_{r}^{2},$$

(6d) $$\tau^{Nq_{0}}\left(n,q\right)=En^{5/3}\ln\left(1+\frac{q_{r}}{q_{0}}\right)=2\tau^{\mathrm{TF}}\beta q_{0}^{2}\ln\left(1+\frac{q_{r}}{q_{0}}\right).$$

In the above equations, $\tau^{\mathrm{TF}}\left(n\right)=3\left(E_{h}a_{0}^{2}\right){\left(3\pi^{2}\right)}^{2/3}n^{5/3}/10\equiv Bn^{5/3}$ is the TF kinetic energy functional, with the Hartree energy $E_{h}=\hbar^{2}/\left(m_{e}a_{0}^{2}\right)$, Bohr radius $a_{0}$, and coefficient $B=3\left(E_{h}a_{0}^{2}\right){\left(3\pi^{2}\right)}^{2/3}/10$. The other coefficients are $A=\lambda_{W}E_{h}a_{0}^{2}/8$, $C={\left(3\pi^{2}\right)}^{-2/3}/4$, $D=3{\left(3\pi^{2}\right)}^{-2/3}E_{h}a_{0}^{2}/160$, and $E=6\left(E_{h}a_{0}^{2}\right){\left(3\pi^{2}\right)}^{2/3}\beta q_{0}^{2}/10$. The reduced Laplacian $q_{r}=3q/\left(40\tau^{\mathrm{TF}}\right)$ is very large for a small density. The parameters α and β are α=40/27 and β=0.25. $q_{0}$ is a parameter that can be used to tune the energy position of the Bennett state. It is $q_{0}=700$ in order to have the same Bennett peak position as that obtained from the TDDFT calculations for a Na jellium nanosphere with 1074 electrons when using DFT density [24].

It should be noted that there is an extra coefficient $\lambda_{W}$ in the expression A compared to that shown in Ref [24]. In that original PGSLN model, this parameter was fixed at $\lambda_{W}=1$. As demonstrated in Ref. [29], the vW term plays a major role that governs the electron spill-out behavior. Small $\lambda_{W}$ leads to less spill-out. Many different studies have explored alternative values, such as $\lambda_{W}=1/9$ [26], $\lambda_{W}=0.5$ [25], and $\lambda_{W}=0.7$ [28]. It is worth noting that these works did not incorporate the Laplacian-level PGSLN functional [25,26,28].

The other undefined quantity in Equation (3) is the XC functional $E_{\mathrm{XC}}\left[n\right]$. As stated in Ref. [26], an exact expression for $E_{\mathrm{XC}}\left[n\right]$ is not known in general, and it is not easy to calculate it numerically either. The most familiar one is the LDA, where no density gradients are considered. The Gunnarson and Lundqvist (GL) LDA XC functional is widely used [26]. The functional derivative with respect to the density *n* reads as follows:

(7) $$\frac{\delta E_{\mathrm{XC}}^{\mathrm{GL}}\left[n\right]}{\delta n}=E_{h}\left[-a_{0}{\left(\frac{3}{\pi }\right)}^{1/3}n^{1/3}-0.0333\ln\left(1+\frac{1}{x}\right)\right],$$

Here, $x={\left(3/4\pi \right)}^{1/3}n^{-1/3}/\left(11.4a_{0}\right)$. Another LDA XC functional is the Perdew–Zunger (PZ) LDA parametrization, which is also used both for Na and Ag [23,24]. It reads as follows:

(8) $$\frac{\delta E_{\mathrm{XC}}^{\mathrm{PZ}}\left[n\right]}{\delta n}=E_{h}\left[-a_{0}{\left(\frac{3}{\pi }\right)}^{1/3}n^{1/3}+\mu_{C}\left[n\right]\right],$$

in which

(9) $$\mu_{C}\left[n\right]=\frac{\alpha_{1}+\frac{7\alpha_{1}\beta_{1}}{6}\sqrt{r_{s}}+\frac{4\alpha_{1}\beta_{2}}{3}r_{s}}{{\left(1+\beta_{1}\sqrt{r_{s}}+\beta_{2}r_{s}\right)}^{2}},$$

with $\alpha_{1}=-0.1423$, $\beta_{1}=1.0529$, $\beta_{2}=0.3334$, and $r_{s}={\left(3/4\pi n\right)}^{1/3}/a_{0}$. In addition, the Wigner (WG) LDA XC functional adopted in Refs. [25,28] on the work function is also considered as follows:

(10) $$\frac{\delta E_{\mathrm{XC}}^{\mathrm{WG}}\left[n\right]}{\delta n}=-\frac{e^{2}}{\varepsilon_{0}}\frac{0.0909a_{0}}{{\left(0.0625+7.8a_{0}n^{1/3}\right)}^{2}}n^{2/3}+\frac{e^{2}}{\varepsilon_{0}}\left(\frac{0.0467}{0.0625+7.8a_{0}n^{1/3}}-0.0783\right)n^{1/3}.$$

Since the XC potential does not have a direct microscopic representation, its explicit form remains ambiguous. In DFT, the choice of XC potential is crucial, as it significantly affects the predicted ground-state properties. However, in the context of QHT, the influence of the XC potential—particularly on ground-state characteristics—remains poorly understood, with limited systematic investigation to date. To address this gap, the present work aims to systematically examine how different forms of the XC potential impact ground-state properties within the QHT framework.

To solve the scattering field formulation within the linearized hydrodynamic model (Equations (1) and (2)), one must first find the ground-state electronic density $n_{0}\left(r\right)$. It can be solved by the static equation of QHT as follows [25,26,28]:

(11) $$\nabla {\left(\frac{\delta G}{\delta n}\right)}_{0}-eE_{0}=0.$$

The static electric field $E_{0}=-\nabla \phi_{0}$ is solved self-consistently with the Poisson equation:

(12) $$\nabla \cdot \left(\varepsilon_{0}\varepsilon_{r}\nabla \phi_{0}\right)=q_{e}\left(n_{b}-n_{0}\right).$$

Here, $n_{b}={\left(4\pi {r_{s}^{+}}^{3}/3\right)}^{-1}$ denotes the positive background charge density, which is uniform inside the metal and zero outside. For sodium, the Wigner–Seitz radius is $r_{s}^{+}=4$.

(13) $${\left(\frac{\delta G}{\delta n}\right)}_{0}-q_{e}\phi_{0}=\mu .$$

Here, μ is the chemical potential. At this time, the vW kinetic energy functional can be regarded as an approximate kinetic energy operator, and the function of vW can be approximated with $\sqrt{n_{0}}$, and the approximate Schrödinger equation can be obtained:

(14) $$\lambda_{W}\frac{\hbar^{2}}{2m}\nabla^{2}\sqrt{n_{0}}+V_{\mathrm{eff}}\sqrt{n_{0}}=\mu \sqrt{n_{0}}.$$

Here, $V_{\mathrm{eff}}={\left(\delta T_{\mathrm{TF}}/\delta n\right)}_{0}+{\left(\delta E_{\mathrm{XC}}/\delta n\right)}_{0}+q_{e}\phi_{0}$. The ground-state density can be obtained by Equations (12) and (14), a method also known as OFDFT. The QHT can also be reformulated within the time-dependent OFDFT framework [27].

## 3. Energy Functional Required for Accurate Ground-State Density in OFDFT

**Figure 1:**
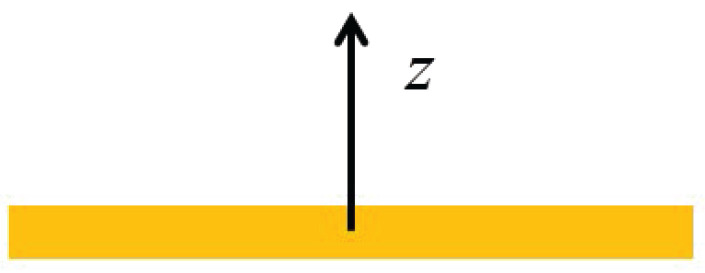
Schematic diagram of the system. The slab is infinite in the xy plane, modeled using a jellium background with $r_{s}^{+}=4$. This study focuses on the ground-state density and work function in the *z* direction.

First, it is necessary to determine the appropriate energy functional for calculating the ground-state density. In this section, we compute the $n_{0}$ distribution of a jellium slab using QHT based on Equations (12) and (14) and compare it with DFT results. For the DFT calculations, the ground-state density of an infinite slab in the xy plane (see Figure 1) was obtained using the open-source software Octopus (version 12.0) [30,31]. The results are stable when the slab thickness in the *z* direction exceeds $L>5\lambda_{F}$, where $\lambda_{F}=3.27r_{s}^{+}$ [32,33].

**Figure 2:**
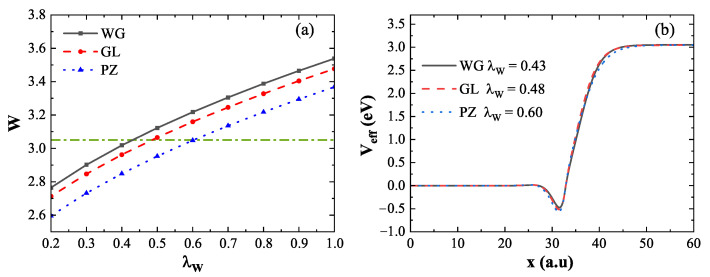
(**a**) Work function as a function of the coefficient $\lambda_{W}$ using WG (black solid line), GL (red dashed line), and PZ (blue dotted line) XC potentials. The horizontal dashed line represents the DFT reference value. The intersection points with the DFT reference are at $\lambda_{W}=0.43$, 0.48, and 0.60, respectively. (**b**) Effective potentials corresponding to these intersections, normalized by μ.

Figure 2a illustrates how the coefficient $\lambda_{W}$ affects the calculated work function when using three representative XC potentials: WG (black solid line), GL (red dashed line), and PZ (blue dotted line). The horizontal dashed line denotes the reference value obtained from the DFT calculation (3.05eV). Taking the WG XC potential as an example, the work function increases from 2.63eV to 3.57eV as $\lambda_{W}$ increases, intersecting the reference line at $\lambda_{W}=0.43$, which is consistent with Ref. [25]. Similarly, the GL and PZ curves intersect the DFT reference line at $\lambda_{W}=0.48$ and 0.60, respectively. These results indicate that matching the DFT work function requires different $\lambda_{W}$ values depending on the chosen XC potential. Figure 2b shows the effective potential $V_{\mathrm{eff}}$, normalized by the chemical potential μ, for the respective $\lambda_{W}$ values determined above. The effective potentials obtained from all three functionals converge around 50a.u., and the corresponding work functions align well with the DFT result. Therefore, to reproduce the DFT-calculated work function, the optimal $\lambda_{W}$ values for WG, GL, and PZ are 0.43, 0.48, and 0.60, respectively. The results demonstrate that the work function is significantly influenced by $\lambda_{W}$: a larger $\lambda_{W}$ leads to a stronger electron spill-out effect and an increase in the work function. Additionally, the choice of XC potential also contributes to variations in the work function.

**Figure 3:**
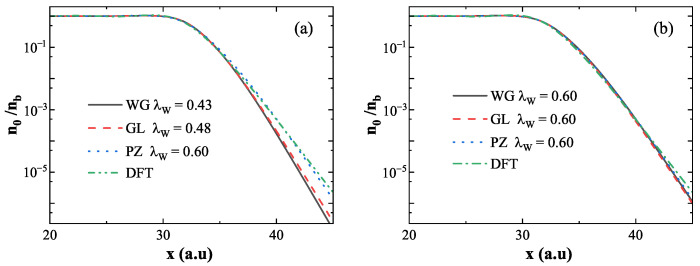
Normalized ground-state density obtained using different energy functionals. (**a**) $\lambda_{W}=0.43$ for WG (black solid line), $\lambda_{W}=0.48$ for GL (red dashed line), and $\lambda_{W}=0.60$ for PZ yield consistent work functions with DFT; DFT result shown in green line. (**b**) WG, GL, and PZ functionals with fixed $\lambda_{W}=0.60$. While $\lambda_{W}$ significantly affects the decay of surface density, the XC potential has negligible influence.

The ground-state electron density near the metal surface plays a critical role in excited-state calculations. Figure 3 presents the normalized ground-state density at the surface of the infinite slab, using $n_{b}$ for normalization. Figure 3a shows the ground-state density computed using three XC functionals, each with a value of $\lambda_{W}$ that reproduces the same work function as obtained by DFT. Specifically, the values used are $\lambda_{W}=0.43$ for WG (black solid line), $\lambda_{W}=0.48$ for GL (red dashed line), and $\lambda_{W}=0.60$ for PZ (blue dotted line). Although these functionals yield consistent work functions, their surface electron densities differ significantly. The density derived from WG ($\lambda_{W}=0.43$) and GL ($\lambda_{W}=0.48$) decays more rapidly near the surface compared to the DFT result, while the PZ ($\lambda_{W}=0.60$) result shows close agreement with DFT. This suggests that the decay rate of the ground-state density is primarily governed by the coefficient $\lambda_{W}$, which modulates the electron spill-out effect. A larger $\lambda_{W}$ leads to greater electron spill-out and a slower decay of surface density. Figure 3b explores the impact of XC potentials on the ground-state density using a fixed $\lambda_{W}=0.60$ for all three functionals. The results show that, under the same $\lambda_{W}$, the choice of XC potential has little influence on the surface electron density. Thus, the surface density is primarily determined by $\lambda_{W}$ rather than the specific form of the XC potential.

These results collectively show that in QHT, the calculated work function is influenced by both $\lambda_{W}$ and the XC potential, while the surface ground-state density is predominantly affected by $\lambda_{W}$ alone. Among the tested functionals, PZ with $\lambda_{W}=0.60$ provides both an accurate work function and a ground-state density profile that best agrees with DFT.

## 4. Energy Functional Required for Accurate Optical Response in QHT

**Figure 4:**
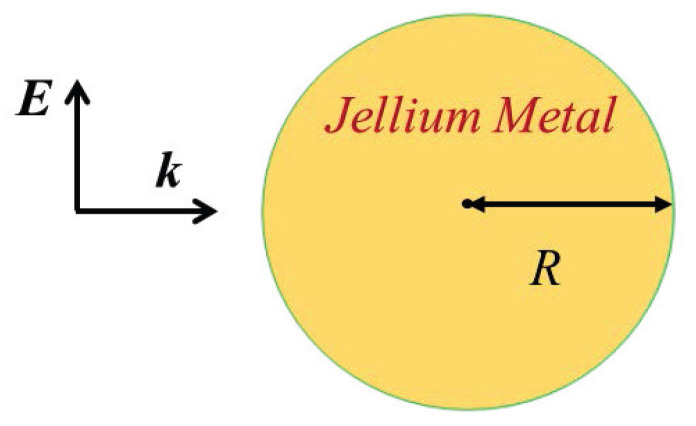
Schematic diagram of the calculation setup. A plane wave is incident perpendicular to the *z*-axis. The absorption cross-section of sodium jellium nanospheres is evaluated using the gel model.

In the previous section, we established that using the PZ with the $\lambda_{W}=0.60$ energy functional within QHT produces work functions and a ground-state density that are consistent with DFT results. In this section, TDDFT results are used as a reference to study the appropriate energy functionals for excited states. We calculate the absorption cross-section (normalized by $\sigma_{0}=\pi R^{2}$) of a jellium sphere under plane wave illumination, as illustrated in Figure 4. The absorption cross-section is given by

(15) σ(ω)=ω2I0∫ImE·P*dV.

Here, E and P are the electric field and polarization, respectively.

### 4.1. Optical Response Calibration by TDDFT

**Figure 5:**
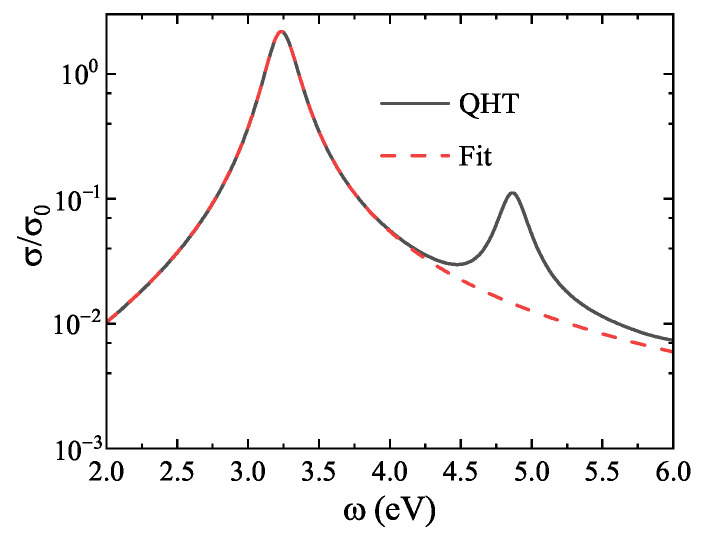
Normalized absorption cross-section (log scale) for a Na jellium nanosphere with 1074 electrons. Solid line: QHT result; dotted line: fitted LSP peak using the method in Ref. [24]. Only the first LSP resonance is considered.

To extract the resonance features, we follow the spectral fitting procedure described in Ref. [24], where only the first localized surface plasmon (LSP) peak is fitted. As an example, Figure 5 shows the normalized absorption cross-section for a nanosphere with 1074 electrons. The fitted curve accurately reproduces the first LSP response.

For excited-state calculations, we use the PGSLN kinetic energy functional. In practice, the LSP response inside the nanosphere is primarily governed by the TFvW component. Therefore, to accurately capture the LSP energy, it is sufficient to determine the appropriate $\lambda_{W}$ parameter in the excited-state functional.

**Figure 6:**
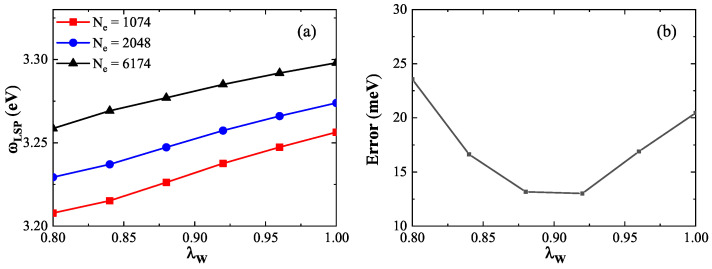
(**a**) LSP energy as a function of the coefficient $\lambda_{W}$ for nanospheres with 1074 (red squares line), 2048 (blue circles line), and 6174 (black triangles line) electrons. All exhibit linear increases in LSP energy with increasing $\lambda_{W}$. (**b**) Average error in LSP energy (relative to TDDFT results) for the three nanospheres. The minimum error occurs near $\lambda_{W}=0.90$.

To compare with the TDDFT benchmark data and ensure consistency with previous work [24], we use 13 jellium nanospheres with electron numbers ranging from 338 to 6174. For calibration, we select three representative nanospheres: a small (1074 electrons), medium (2048 electrons), and large (6174 electrons) sphere. Figure 6a shows how the LSP energy varies with the coefficient $\lambda_{W}$ for the three spheres. The results indicate that the LSP energy increases approximately linearly with $\lambda_{W}$. For instance, in the 1074-electron sphere, increasing $\lambda_{W}$ from 0.80 to 1.00 raises the LSP energy from 3.21eV to 3.26eV, yielding a linear slope of 0.25eV. For the 2048- and 6174-electron spheres, the slopes are 0.21eV and 0.29eV, respectively. These results demonstrate that the LSP energy is sensitive to $\lambda_{W}$—a higher $\lambda_{W}$ results in greater LSP energy due to enhanced electron spill-out. However, the slope of this increase is smaller for larger spheres, suggesting that the spill-out effect becomes less significant as the system size increases. Figure 6b presents the average relative error in LSP energy (compared to TDDFT results) for the three nanospheres as a function of $\lambda_{W}$. The minimum error occurs between $\lambda_{W}=0.88$ and 0.92, with both yielding comparable accuracy. To maintain consistency with the ground-state calibration, $\lambda_{W}=0.90$ is selected for subsequent excited-state calculations.

### 4.2. LSP Energy and Corresponding Oscillator Strength of Different Sizes

**Figure 7:**
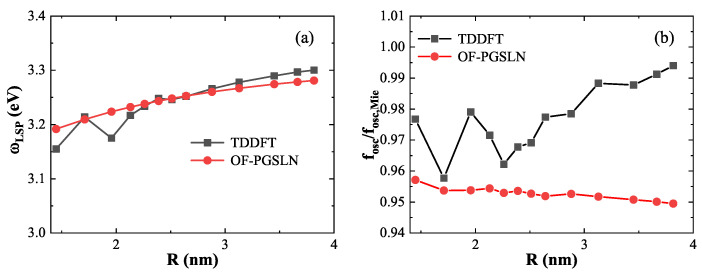
(**a**) LSP energy and (**b**) corresponding oscillator strength (normalized to the classical Mie results) for nanospheres of various sizes calculated by OF-PGSLN (red lines) compared with TDDFT reference values (black lines). The OF-PGSLN results show excellent agreement with TDDFT across the full range of nanosphere diameters.

In the previous section, the excited-state energy functional was calibrated using three representative nanospheres. To further assess the accuracy and generalizability of our approach across a broader size range, Figure 7 presents the LSP energies and corresponding oscillator strengths for nanospheres of various sizes, as computed using the OF-PGSLN method (red dashed lines) and compared with TDDFT results (black solid lines) from Ref. [24].

Figure 7a shows the LSP energies for nanospheres containing 338 to 6174 electrons. The OF-PGSLN results exhibit excellent agreement with TDDFT, with an average absolute error of 0.014eV. This is comparable to the error associated with the model ground-state density reported in Ref. [24] although slightly larger than that obtained using the DFT ground-state density (0.006eV). Figure 7b displays the corresponding oscillator strengths, normalized by the classical local-response approximation value $f_{\mathrm{osc},\mathrm{Mie}}$. The mean error for OF-PGSLN is 0.021 across 13 nanospheres, which lies between the error obtained with the DFT ground-state density (0.029) and that with the model ground-state density (0.008).

These results confirm that the OF-PGSLN approach yields LSP energies and oscillator strengths with accuracy comparable to that of methods that rely on DFT or model ground-state densities, despite employing an OFDFT ground-state framework. By directly optimizing both ground- and excited-state energy functionals, OF-PGSLN consistently reproduces TDDFT results with similar mean errors. This self-contained formulation eliminates the dependence on DFT, making OF-PGSLN a flexible and computationally efficient alternative, particularly well-suited for large or complex plasmonic systems.

## 5. Validity and Limitations of Linear Superposition Approximation in Nanodimers

In the previous section, we demonstrated that the OF-PGSLN ground-state density enables accurate predictions of both the LSP energy and oscillator strength in sodium nanospheres. We now extend this analysis to a dimer system composed of two sodium jellium spheres, each containing 1074 electrons. This system provides a platform for evaluating the validity of a linear superposition of ground-state density across varying interparticle distances. Specifically, we assess the performance of the OFDFT-derived ground-state density in comparison with that obtained from a linear superposition of model ground-state density for different gap sizes. The model ground-state density is defined as $n_{\mathrm{mod}}\left(r\right)=f_{0}/\left\{1+\exp[\kappa (r-R)]\right\}$, where $\kappa =1.05/a_{0}$ and $f_{0}=0.98n_{b}$. Here, R denotes the radius of the nanosphere and *r* represents the radial distance from the center of the sphere [24].

**Figure 8:**
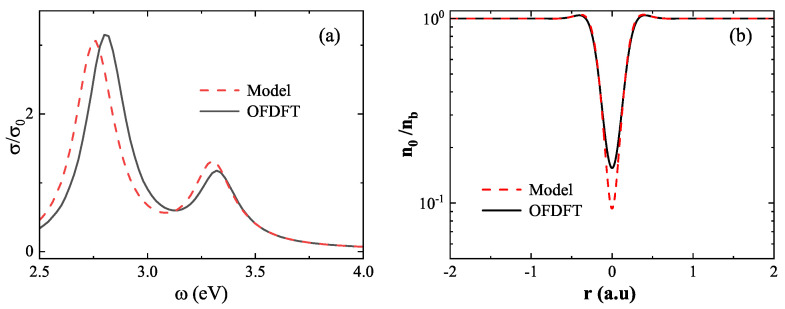
Breakdown of the linear superposition approximation at short distances (gap=0.3nm). (**a**) Absorption spectra computed using model density (red dashed line) and OFDFT density (black solid line), showing a significant redshift in the LSP peak when using the linear superposition. (**b**) Ground-state electron density at the dimer center, where the linear superposition approximation underestimates the density due to neglected quantum effects.

We begin with the small-gap regime, where strong quantum effects emerge due to significant wavefunction overlap. As shown in Figure 8a, at gap=0.3nm, the absorption spectrum based on the model density (red dashed line) exhibits a marked redshift in the first LSP peak relative to the spectrum obtained using the OFDFT density (black solid line), with a difference of 56meV. To elucidate the source of this discrepancy, Figure 8b presents the ground-state electron density at the center of the dimer. The OFDFT result yields a minimum density of $1.5\times 10^{-1}$, whereas the model result gives a significantly lower value of $9.3\times 10^{-2}$. This underestimation arises from the model’s neglect of essential quantum mechanical effects—namely, electron–electron repulsion, Fermi pressure, and quantum confinement—which become critical in the strongly coupled regime. These findings reveal the breakdown of the linear approximation at short distances and emphasize the necessity of using the OFDFT ground-state density when modeling closely spaced nanostructures.

**Figure 9:**
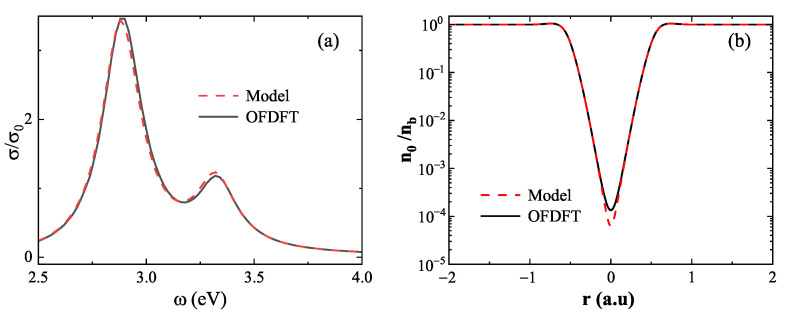
Validation of the linear superposition approximation at large distances (gap=1.0nm). (**a**) Absorption spectra computed from model density (red dashed line) and OFDFT density (black solid line) show excellent agreement. (**b**) Ground-state electron density at the dimer center, revealing only a minor difference between the two methods.

In contrast, the large-gap regime corresponds to a situation in which the electron density of the two nanospheres remains largely non-overlapping, and mutual interactions are minimal. As illustrated in Figure 9a, at gap=1.0nm, the absorption spectra calculated from both the model density (red dashed line) and the OFDFT density (black solid line) are nearly identical, differing by only 6meV in LSP energy. This agreement is further supported by Figure 9b, which shows the minimum electron density at the dimer center: $6.38\times 10^{-5}$ for the model density and $1.39\times 10^{-4}$ for the OFDFT density. The small discrepancy confirms the validity of the linear superposition approximation in the weakly coupled regime. Notably, this approximation offers a practical advantage: for systems comprising many nanostructures, individual ground-state densities can be precomputed and linearly combined, significantly reducing computational cost without sacrificing accuracy.

**Figure 10:**
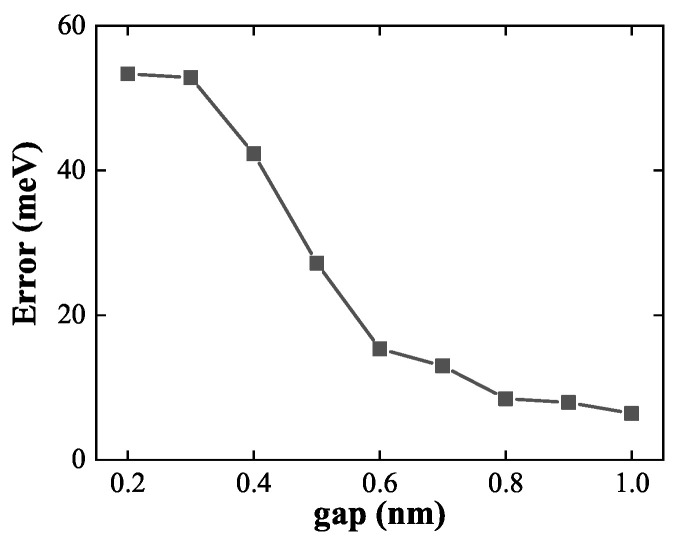
LSP energy error between model and OFDFT density as a function of dimer gap. The error remains small at large separations but increases rapidly below 0.6nm, signaling the breakdown of the linear superposition approximation in the strong coupling regime.

To quantitatively evaluate the performance of the linear approximation across different interparticle separations, Figure 10 plots the LSP energy error as a function of dimer gap. While the error remains below 10meV for gaps larger than 0.8nm, it increases sharply below 0.6nm, saturating at approximately 54.2meV in the strongly coupled regime. These results highlight the dual nature of the linear superposition approach: it fails under strong coupling but becomes a powerful and efficient modeling tool when the constituent nanostructures are sufficiently separated. This strategy is especially advantageous for simulating large nanoparticle assemblies, where reusing precomputed ground-state density can drastically reduce computational overhead.

## 6. Conclusions

In this work, we developed OF-PGSLN, a QHT framework that integrates OFDFT for ground-state density with a Laplacian-level kinetic energy functional (PGSLN) for excited-state dynamics. The key conclusions are as follows: Using DFT as a benchmark, we determined that the PZ XC functional with $\lambda_{W}=0.60$ yields the most accurate ground-state properties, including work functions and surface electron density. For excited states, calibrating against TDDFT, we found that $\lambda_{W}=0.90$ enables precise reproduction of LSP resonances. Across sodium nanospheres with $338\leq N_{e}\leq 6174$, the average errors in resonance energy and oscillator strength are only 0.014eV and 0.021, respectively. We further applied the method to nanodimers, demonstrating that while the linear superposition model suffices at large gaps (>0.8 nm), it fails to capture critical density redistribution at small gaps (<0.6 nm), leading to significant errors—up to 56meV—which OF-PGSLN accurately resolves. By enabling stable, geometry-independent ground-state calculations and suppressing spurious spectral features through PGSLN, our approach provides a reliable and efficient platform for modeling quantum optical responses in complex nanostructures, with broad applicability to next-generation plasmonic and nanophotonic devices.

## Data Availability

The original contributions presented in this study are included in the article. Further inquiries can be directed to the corresponding author(s).
